# Mode of Glucocorticoid Actions in Airway Disease

**DOI:** 10.1100/tsw.2006.274

**Published:** 2006-12-28

**Authors:** Kazuhiro Ito, Steve Getting, Catherine Charron

**Affiliations:** Airways Disease Section National Heart and Lung Institute, Imperial College London, London, UK

**Keywords:** glucocorticoid, inflammation, asthma, COPD, glucocorticoid receptor, glucocorticoid resistance

## Abstract

Synthetic glucocorticoids are the most potent anti-inflammatory agents used to treat chronic inflammatory disease, such as asthma. However, a small number (<5%) of asthmatic patients and almost all patients with chronic obstructive pulmonary disease (COPD) do not respond well, or at all, to glucocorticoid therapy. If the molecular mechanism of glucocorticoid insensitivity is uncovered, it may in turn provide insight into the key mechanism of glucocorticoid action and allow a rational way to implement treatment regimens that restore glucocorticoid sensitivity. Glucocorticoids exert their effects by binding to a cytoplasmic glucocorticoid receptor (GR), which is subjected to post-translational modifications. Receptor phosphorylation, acetylation, nitrosylation, ubiquitinylation, and other modifications influence hormone binding, nuclear translocation, and protein half-life. Analysis of GR interactions to other molecules, such as coactivators or corepressors, may explain the genetic specificity of GR action. Priming with inflammatory cytokine or oxidative/nitrative stress is a mechanism for the glucocorticoid resistance observed in chronic inflammatory airway disease via reduction of corepressors or GR modification. Therapies targeting these aspects of the GR activation pathway may reverse glucocorticoid resistance in patients with glucocorticoid-insensitive airway disease and some patients with other inflammatory diseases, such as rheumatoid arthritis and inflammatory bowel disease.

### INTRODUCTION

Although glucocorticoids are the most potent anti-inflammatory agents for the treatment of chronic inflammatory disease, such as asthma, a small population of asthmatic patients and almost all patients with chronic obstructive pulmonary disease (COPD) show a poor response to glucocorticoids[1,2]. These patients present considerable management problems and account for a huge part of expenditure of the health care costs for airway diseases. In particular, treatment of severe asthma accounts for 50% of the health care cost for asthma. COPD is a major and increasing global health problem[3] and is predicted to become the third most common cause of death and the fifth most common cause of chronic disability worldwide by 2020[4]. If the molecular mechanisms for glucocorticoid insensitivity are better understood, they might provide new insight into the modes of glucocorticoid action and allow a rational way to treat the patients whose disease tends to be severe. Here we summarize the molecular mechanisms of glucocorticoids as related to airway diseases.

### THE MOLECULAR BASIS OF INFLAMMATION IN ASTHMA AND COPD

Lower airways inflammation is a central feature of many lung diseases including asthma and COPD. These diseases always involve recruitment and activation of inflammatory cells with changes in the structural cells of the lung, though the specific characteristics of the inflammatory response and the site of inflammation differ from one disease to another. Inflammation in asthma is associated with increased airway hyper-responsiveness leading to recurrent episodes of wheezing, breathlessness, chest tightness, and coughing, particularly at night or in the early morning. The inflammation is present even in those with very mild asthma; T-lymphocytes of the T-helper (Th) type 2 phenotype, eosinophils, macrophages/monocytes, and mast cells infiltrate the airway wall. Airway inflammation is also amplified during exacerbation with an increase in eosinophils and sometimes neutrophils. These conditions are also characterized by an increased expression of components of the inflammatory cascade including chemokines, cytokines, growth factors, enzymes, noxious gas, reactive oxygen, receptors, and adhesion molecules[5].

COPD is a chronic inflammatory disease of the lower airways and lung, which is enhanced during exacerbations[6,7]. The pathological characteristics of COPD are destruction of the lung parenchyma (emphysema), inflammation of the peripheral and central airways, and increased mucus-producing cells[8]. Most patients with COPD show chronic obstructive bronchitis, emphysema, and/or mucus plugging. There is a marked increase in macrophages and neutrophils in bronchoalveolar-lavage fluid and induced sputum, and T cells and B-lymphocytes in lung parenchyma. COPD is also characterized by an increased expression of many mediators involved in the inflammatory cascade including cytokines, chemokines, growth factors, enzymes, lipid mediators, receptors, and adhesion molecules[9]. Notably, increased production of tumor necrosis factor-α (TNFα), interferon-γ (IFNγ), interleukin-8 (IL-8), macrophage inflammatory protein 1α (MIP-1α), GRO-α, monocyte chemoattractant protein I (MCP-1), leukotrien $B_4$, and matrix metalloproteinase (MMP)-9 are found in COPD[10].

Increased inflammatory gene transcription seen in inflammatory airway disease is regulated by proinflammatory transcription factors, such as nuclear factor-κB (NF-κB) and activator protein-1 (AP-1). NF-κB is ubiquitously expressed and is able to not only control the induction of inflammatory genes in its own right, but also enhances the activity of other cell- and signal-specific transcription factors[11]. In addition, it is a major target for glucocorticoids[12,13,14]. AP-1 is a transcription factor complex that is formed by dimerization of members of the *Fos* (c-Fos, Fra1, and Fra2) and *Jun* (c-Jun, Jun B, and Jun D) proto-oncogene families and is defined by binding to the phorbol ester 12-O-tetradecanoylphorbol-13-acetate (TPA)-response element (TRE), and it is also a major target for glucocorticoids[12,15]. There is increasing evidence that these transcriptional factors are activated in asthma and COPD[16].

Alterations in the structure of chromatin are critical to the regulation of gene expression[17] as shown in Fig. 1. The chromatin structure is composed of nucleosomes, which are particles consisting of ~146-bp DNA associated with an octamer of two molecules each of core histone proteins (H2A, H2B, H3, and H4). In the resting cell, DNA is tightly compacted around these basic core histones, excluding the binding of the transcriptional factors and the enzyme RNA polymerase II, which activates the formation of messenger RNA. This conformation of the chromatin structure is described as closed and is associated with suppression of gene expression. Acetylation of lysine residues on histones induces a relaxed DNA structure, allowing transcription factors to access the DNA and induce gene transcription. Transcriptional coactivators, such as CREB (cyclic AMP response element binding protein ) binding protein (CBP), have intrinsic histone acetyltransferase (HAT) activity, which is further activated by the binding of transcription factors. Changes in the phosphorylation status of HATs affect their activity. Increased gene

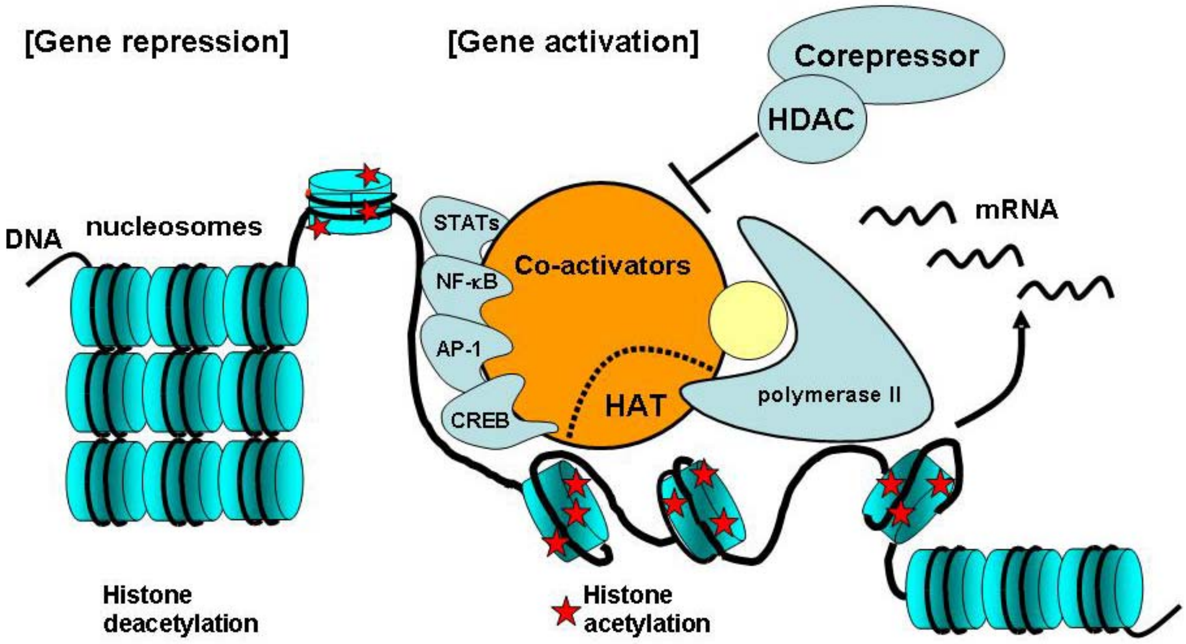

**FIGURE 1**. Molecular mechanism of gene activation and repression. Gene activation and repression are regulated by acetylation of core histones. Histone acetylation is mediated by coactivators that have intrinsic HAT activity, opening up the chromatin structure to allow binding of RNA polymerase II and transcription factors that were unable to bind DNA in the closed chromatin configuration. This is reversed by corepressors, which include histone deacetylases (HDACs) and other associated proteins that reverse this acetylation, thereby causing gene silencing. STATs, signal transduction activated transcription factors.

transcription is, therefore, associated with an increase in histone acetylation, whereas hypoacetylation is correlated with reduced transcription or gene silencing, which is regulated by histone deacetylase (HDAC)[18]. As well as the acetylation of histones, phosphorylation, methylation, and other modifications on histones are also involved in the epigenetic control of gene expression[19].

### MECHANISMS OF GLUCOCORTICOID RECEPTOR FUNCTION

Glucocorticoids exert their effects by binding to a single 777-amino-acid glucocorticoid receptor (GR) that is localized to the cytoplasm of almost all cell types[20,21]. GR has several functional domains as shown in Fig. 2. The glucocorticoid ligand binding domain (LBD) is at the carboxyl terminus of the molecule and contains the motif for ligand-dependent transcriptional activation function 2 (AF2), and this site is also involved in protein-protein interactions with either cytosolic chaperones or cofactors. The DNA binding domain (DBD), a highly conserved zinc finger is separated from the carboxyl end by a hinge region. The N-terminus encodes the constitutive transcription activation function 1 (AF1). Inactive GR is part of a large protein complex (~300 kDa) that includes two subunits of the heat shock protein hsp90, acting as molecular chaperones preventing the nuclear localization of unoccupied GR, and immunophilins, such as FKBP1. Once the ligand binds to GR, hsp90 dissociates, allowing the nuclear localization of the activated GR-steroid complex via association with import proteins, such as importin α[22].

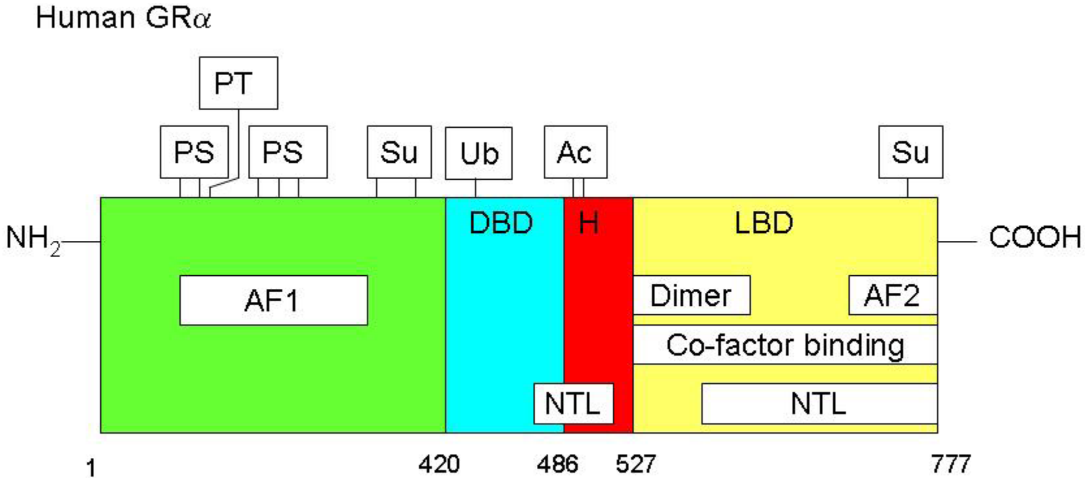

**FIGURE 2.** Structure of GRα and its post-translational modification. The N-terminus represents the constitutive transcriptional activation function (AF-1). The C-terminus contains the LBD, ligand-dependent activation function (AF-2), dimerization domain, and cofactor binding domains, all separated from the DBD by the hinge region (H). GR is post-translationally modified. Su (sumolyation), Ub (ubiquitinylation), PS (serine phosphorylation), PT (threonine phosphorylation), Ac (acetylation).

### HOW TO SWITCH OFF INFLAMMATORY GENES

The major anti-inflammatory effects of glucocorticoids are thought to be due to repression of inflammatory and immune genes[23]. The GR complex may regulate gene expression in at least five different ways (Fig. 3). First, GR acting as a monomer can bind directly or indirectly with the transcription factors AP-1 and NF-κB. As discussed above, in chronic inflammation, there is a coordinated expression of multiple inflammatory genes including cytokines, chemokines, adhesion molecules, and inflammatory enzymes that have been activated by NF-κB and AP-1. Classically, activated GR was known to interact with these proinflammatory transcriptional factors, not allowing them to bind to specific binding sites of DNA and consequently preventing the stimulation of inflammatory gene expression[24]. More recently, the interplay between transcription factors and GR is thought to reflect differing effects on histone acetylation/deacetylation[25]. That is, this increase in gene expression is brought about by acetylation of core histones as described earlier. Glucocorticoids reverse this process by reversing histone acetylation through the recruitment of HDAC2 to the activated coactivator complex[17]. This then results in rewinding and compacting of DNA, the exclusion of RNA polymerase, and the suppression of inflammatory gene transcription. This mechanism can account for the anti-inflammatory effect of glucocorticoids in asthma and other inflammatory diseases. In fact, in bronchial biopsies of untreated asthmatic patients, there is an increase in HAT activity that is reversed in patients controlled on inhaled corticosteroids[26].

Second, the GR represses gene expression via DNA binding. After GR nuclear translocation, GR combines with another GR to form a dimer and it binds to consensus DNA sites termed glucocorticoid response elements (GREs, GGTACAnnnTGTTCT) in the regulating regions of corticosteroid-responsive genes. The GR dimer can bind to a GRE that overlaps the DNA binding site for a proinflammatory transcription factor or at the start site of transcription such as in the genes for IL-6 and osteocalcin, thus blocking gene expression[1]. This element is called negative GRE (nGRE). It is likely that glucocorticoid-induced side effects, such as osteoporosis, glaucoma, growth retardation in children, wound healing, and metabolic effects, are mediated, at least in part, by DNA binding[27].

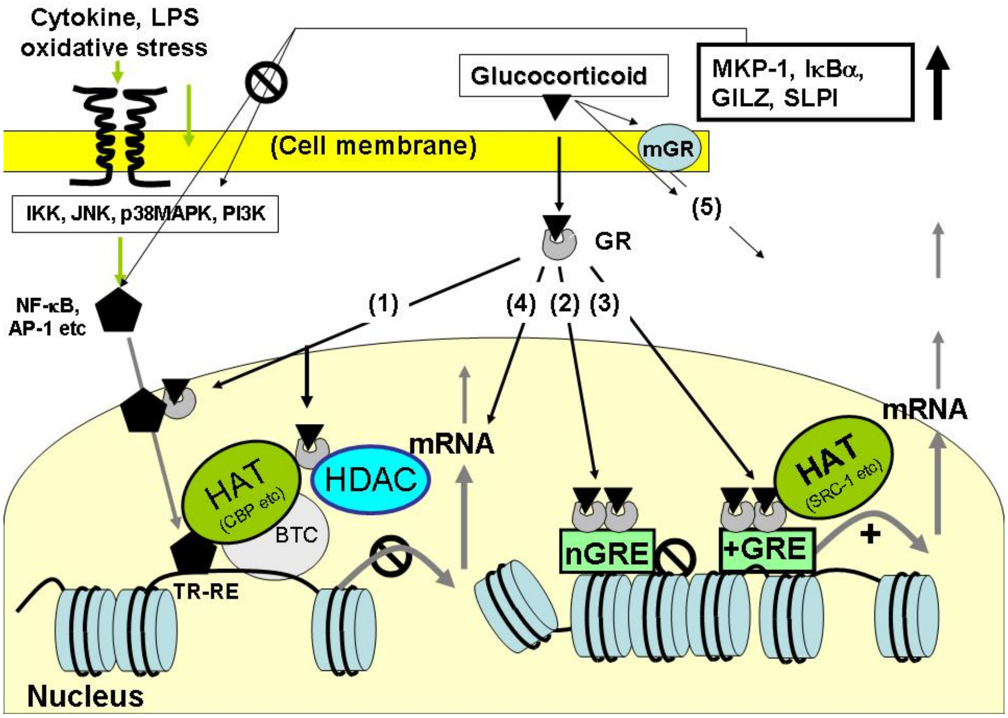

**FIGURE 3.** How glucocorticoids turn off gene transcription. Cytokine, oxidative stress, growth factors, and lipopolysaccharides (LPS) stimulate activation of transcriptional factors, such as NF-κB. Activated NF-κB translocates to nuclei and recruits HAT, and then acetylates histones. Consequently, genes are switched on as shown in the left side. GR exists in the cytoplasm (center of figure). After ligand binding, GR translocates into nuclei. (1) GR monomers associate with transcription factors-HAT complex, and finally recruit HDAC, which subsequently deacetylases histone and switches off the gene. (2) The GR dimer can bind negative-GRE to suppress transcription of some genes, such as IL-6, directly or (3) bind the promoter region of anti-inflammatory factors (+GRE) and activate them. In addition, glucocorticoid-GR complex is involved in reduced mRNA stability (4) and reduced translation of proinflammatory cytokines. Furthermore, GR may act directly (nonspecifically) or via membrane bound GR (mGR) (5).

Third, the GR dimer, through the binding to GRE, may suppress inflammation by increasing the synthesis of anti-inflammatory proteins, such as IL-10, MAPK phosphatase-1 (MKP-1), the inhibitor of NF-κB (IκB-α), annexin 1 (and annexin 1 peptides), glucocorticoid-induced leucine zipper (GILZ), and secretory lymphocyte protease inhibitor (SLPI)[1]. This interaction allows GR to associate with regulatory proteins, including coactivators, which produce a DNA-protein structure that allows enhanced gene transcription through an action on chromatin remodeling and recruitment of RNA polymerase II to the site of local DNA unwinding via histone acetylation or other epigenetic modification[25,28,29].

Fourth, glucocorticoids can increase the levels of cell ribonucleases and mRNA destabilizing proteins, thereby reducing the levels of mRNA[30] (Fig. 3). It is likely that the altered translation of many different genes is involved in the anti-inflammatory actions of glucocorticoids[22]. An alternative mechanism for GR-p65 cross-talk has been proposed by Nissen and Yamamoto[31] who clearly show that dexamethasone acts by repressing the phosphorylation of RNA polymerase II CTD, thereby altering the ability of RNA polymerase II to elongate.

Last, GR is also reported to act on the cell membrane nonspecifically or through membrane-bound GR. This is known as nongenomic effects of glucocorticoids, but it is not fully elucidated[32].

### GLUCOCORTICOID RECEPTOR MULTICOMPLEXES WITH VARYING COFACTORS

Activation of GR by glucocorticoids is a multistep process that involves hormone binding, hormone-dependent structural transformation, translocation into the nucleus, and identification of target genes. There is increasing evidence that each step in this process is controlled by the interaction of GR with cofactors, such as a heat shock protein 90 (hsp90)-containing chaperone complex, kinases, high mobility group (HMG) proteins, mineralocorticoid receptor, and other transcription factors[33,34,35]. Additionally, ligand-dependent transcriptional activation/repression appears to depend on its interaction with either coactivators or corepressors, which regulate the structure of chromatin and the recruitment of the basal transcriptional machinery[17,25,36,37]. Coactivators/corepressors are often part of multiprotein complexes that are not specific for GR, but mediate the activity of other nuclear receptors (NRs) and unrelated transcription factors. Surprisingly, recent results reveal that the activity of coactivators might contribute to the receptor, promoter, and cell specificity of NR action. However, the molecular basis underlying this specificity remains largely unknown.

Components of the BRG1(SW1/SNF) complex, an ATP-dependent chromatin remodeling complex, are the well-known coactivators associated with GR[38,39]. This complex potentiates the activity of GR in yeast and mammalian cells.

CBP and p300 are histone acetylases and act as scaffold molecules that interact stably or transiently with a large number of transcription factors, including GR. CBP/p300 is recruited to both GR and other NRs either directly, through AF-1, or indirectly through coactivators interacting with AF-2 and this is involved in GR transactivation[40,41,42]. The PCAF (p300/CBP-associated factor) complex was also shown to be important for GR-dependent transcriptional activation in yeast and mammalian cells and has HAT activity to regulate chromatin structure. These complexes appear to play overlapping roles in AF-1–mediated transcriptional regulation and can compensate for one another under certain conditions. The PCAF complex is also recruited to NRs through interaction with CBP/p300 and p160 coactivators[43,44].

Other well-known GR-associated coactivators are the molecules in the p160 coactivator family[36,41,44,45]. The family has three members: SRC-1 (nuclear receptor coactivator-1/NCOA-1), SRC-2 (transcriptional intermediary factor 2 [TIF2]/glucocorticoid receptor interacting protein 1 [GRIP-1]/nuclear receptor coactivator-2 [NRC-2]), and SRC-3 (acetyltransferase/p300/CBP-interacting protein/receptor-associated coactivator 3 [RAC3]/thyroid hormone receptor-activated molecule 1[TRAM1]/amplified in breast cancer 1 [AIB1]). Multiple LXXLL motifs (where L is leucine and X is any amino acid) contained in the SRCs are responsible for ligand-dependent interaction with NRs in AF-2. These coactivators are also HATs that interact transiently with a large number of NRs; additional functions of the carboxyl termini of SRCs are to bind other coactivators, such as the coactivator-associated arginine methyltransferase-1 (CARM1), and to provide intrinsic HAT activity in SRC-1 and SRC-3[46,47,48].

Li et al. showed that upon ligand treatment, progesterone receptor (PR) interacted preferentially with SRC-1, which recruited CBP and significantly enhanced acetylation at K5 of histone H4[49]. In contrast, activated GR preferentially associated with SRC-2, which subsequently recruited PCAF and led to specific modification of histone H3, suggesting that specific coactivators recruit distinct HAT to modulate the transcription of glucocorticoid-responsive genes. However, we showed that activated GR also induced acetylation of lysine 5 on histone 4, suggesting GR might recruit SRC-1 to its complex[25,50]. In fact, SRC-1 recruitment to the GR complex is shown[51,52].

Very interestingly, the GR coactivators, SRC-1 and GRIP-1 (SRC-2), also act as corepressors of the GR[52,53,54] so are versatile cofactors. For example, GRIP1 competes with IRF3 (interferon gamma regulatory factor 3)-mediated transcription[53]. PELP1 (proline-, glutamic acid-, and leucine-rich protein 1) also serves as a corepressor of GR, although it has been recognized as a coactivator of estrogen receptor (ER), recruiting p300/CBP to the target chromosome[55].

Real corepressors are also recruited to the GR multicomplex, such as SMRT (silencing mediator for retinoid and thyroid-hormone receptors), NCoR (nuclear receptor corepressor), and receptor-interacting protein 140 (RIP140)[56,57,58,59], which negatively regulate the ligand-induced activity of the GR. Significantly, the effects of TIF2 (SRC-2) and SMRT are mutually antagonistic. Wang et al. showed that RU486, a GR antagonist, recruits NCoR to the C-terminal at tyrosine 735, while dexamethasone, a GR agonist, recruits SRC-1, suggesting that different synthetic compounds induce different cofactor recruitment[59]. Therefore, the selection of corepressor and coactivator associated with GR is crucial for the specificity of GR action and ligands seem able to achieve this. Very interestingly, Coqhlan et al. synthesized a compound, AL-438, which reduces the interaction between GR and PGC-1 (peroxisomal proliferator-activated receptor gamma coactivator-1), a cofactor critical for steroid-mediated glucose up-regulation, while maintaining normal interactions with GRIP-1[60]. This compound may be a new type of dissociated glucocorticoid with anti-inflammatory effects, but less GR-dependent side effects.

HDAC2 is the most important corepressor to determine glucocorticoid insensitivity[17,25]. Knock down (KD) of HDAC2 by RNA interference decreased glucocorticoid sensitivity in GR-dependent transrepression in culture cells and sputum macrophages from healthy volunteers, though it enhanced GR-dependent transcriptional activation[61]. In addition, overexpression of HDAC2 restored glucocorticoid sensitivity in alveolar macrophages of COPD patients, who have less HDAC2 expression, and glucocorticoid insensitivity[61]. Thus HDAC2 is viewed as a corepressor, but Qiu et al. have shown that HDAC1, when acetylated, serves as a coactivator for the GR[62]. The role of cofactors in the multiple complexes of GR is not fully understood and further basic research is still required.

### POST-TRANSLATIONAL MODIFICATION

#### Phosphorylation

GR is a phosphoprotein containing multiple potential sites for phosphorylation[63]. The receptor is constitutively phosphorylated under physiological condition and also undergoes an agonist-induced or cell cycle–dependent hyperphosphorylation. The role of receptor phosphorylation in receptor function is controversial; however, promoter complexity and context may affect the ability of phospho-GR to regulate transcription.

Eight phosphorylation sites (seven for serine, one for threonine) in mouse GR have been identified[64]. All phosphorylated residues are clustered in the N-terminal region of the receptor (Fig. 2). Among them, two sites, Ser224 and Ser232 (corresponding to Ser203 and Ser211 in human GR[65]), are phosphorylated to a greater extent in the presence of hormone. Differentially phosphorylated receptor species are located in unique subcellular compartments, likely modulating a distinct aspect of receptor function. Evidence clearly suggests that altered GR phosphorylation status can affect GR ligand binding in nuclei[66], hsp90 interactions[67], subcellular localization[68,69], nuclear-cytoplasmic shuttling[70,71], and transactivation potential[68], possibly through association with coactivator molecules[69]. On the other hand, there are some reports showing that phosphorylation elicited only little/no effect on GR mRNA expression, nuclear translocation/subnuclear localization, and transcriptional activation[72,73,74].

Bodwell et al. showed that GR response varies during the cell cycle, with cells being less sensitive to corticosteroids during G2/M, and in the G2/M period, GR was highly phosphorylated[75]. This suggests phosphorylation of GR may be one of the molecular mechanisms of glucocorticoid resistance. We also preliminarily found that GR is highly phosphorylated in peripheral blood mononuclear cells (PBMCs) from glucocorticoid-insensitive severe asthmatics and in IL-2/IL-4–treated glucocorticoid-insensitive U937 cells (manuscript in preparation). In addition, recent evidence suggests that GR phosphorylation is involved in receptor turnover and that phosphorylation can target the receptor for hormone-mediated degradation[76]. As such, phosphorylation-induced targeting of GR for ubiquitination and proteasomal degradation may play an important role in overall GR responsiveness. Major kinases are responsible for the receptor phosphorylation, such as MAPK, CDK, GSK-3, and JNK, and their activation or overexpression can also target specific serine/threonine residues in GR, decreasing GR-mediated transactivation[63,66,77].

#### Acetylation

HAT and HDAC, which are associated with GR, are also known as protein acetyltransferase/deacetylase as well as histone acetyltransferase/deacetylase. Previous studies have shown that both the estrogen receptor

(ER) and the androgen receptor (AR) are acetylated within their hinge/LBDs and that this can modulate hormone-induced gene induction[78,79]. This NR acetylation site is conserved among members of related NRs and, based on these findings[80], there is a potential acetylation site at aa492-495 (KTKK) within the DBD/hinge region of GR (Fig. 2). Indeed, we showed that GR is acetylated following dexamethasone binding and the acetylation levels were decreased when lysines 494 and 495 on GR were mutated to alanine (A), asparagine (N), or glutamine (Q)[61]. These mutants did not further induce SLPI expression by dexamethasone, although overexpression of native GR enhanced SLPI expression by dexamethasone. There was no difference in ability of native or mutant GR to bind to p65-NF-κB. However GR-mediated suppression of IL-1β–stimulated granulocyte-macrophage colony-stimulating factor (GM-CSF) release was not affected by trichostatin A (TSA), an HDAC inhibitor, with the K494 and K495 mutants, although the repression was attenuated by TSA in native GR-overexpressing cells. This suggests that GR acetylation negatively regulates dexamethasone-induced repression of NF-κB–dependent gene expression. In addition, we found that p65-NF-κB–associated GR was deacetylated. Since in knocked-down cells GR is unable to associate with the p65-NF-κB complex in HDAC2, this deacetylation must be important for GR-mediated transrepression (Fig. 4). Importantly, HDAC2 KD does not inhibit GR-GRE binding or SLPI transactivation, indicating that the acetylated GR is still able to activate glucocorticoid-responsive genes, which may be involved in some of the deleterious side effects that limit the clinical use of these powerful drugs as well as some minor anti-inflammatory effects via induction of anti-inflammatory molecules such as SLPI and MKP-1. Actually, we showed that acetylated GR was clearly a substrate for HDAC2, although it is also partially deacetylated by HDAC 3[61]. Thus, acetylated GR is a substrate of HDAC2, and deacetylation of GR by HDAC2 may be a prerequisite for GR association with the p65-NF-κB–activated complex with subsequent suppression of inflammatory gene expression. This mechanism provides a molecular explanation for the ability of GR to distinguish between recruitment of coactivator and corepressor proteins as previously demonstrated for GRIP-1[54] and the subsequent ability to transactivate or repress gene transcription.

We previously reported that HDAC2 expression and activity is decreased in smokers[81] and patients with COPD[82] who are known to be insensitive to the anti-inflammatory effects of glucocorticoids[83]. Acetylation levels in immunoprecipitated nuclear GR after dexamethasone ($10^{-8}\,M$) treatment were increased in alveolar macrophages obtained from patients with COPD[61]. GR hyperacetylation due to a defect of HDAC might be one of the molecular mechanisms of glucocorticoid resistance seen in COPD.

### Nitrosylation/Nitration

Oxidative/nitrative stress is the key factor in pathogenesis of COPD and maybe in severe asthma[84,85]. Actually, NO or $H_2O_2$ are reported to be increased in exhaled gas and their metabolite or footprint, such as nitrite/nitrate, nitro-tyrosine, and 8-isoprostane, were found in sputum, exhaled breath condensate, and bronchoalveolar lavage from patients with COPD and asthma. Peroxynitrite is produced by the reaction between active oxygen and NO, and is known to nitrate/nitrosylate or oxidize proteins. These modifications on proteins will alter their function and stability. Cysteine residues of GR are targets of *S*-nitrosylation. In fact, Galigniana et al. showed that NO donors (*S*-nitroso-acetyl-DL-penicillamine, *S*-nitroso-DL-penicillamine, or *S*-nitroso-glutathione) decreased the number of ligand binding sites and $K_d$ for the binding of [$^3$H]-triamcinolone to immunoprecipitated GR from mouse L929 fibroblasts, without any change in GR protein levels[86]. GR is also a nitrated protein. Interestingly, the addition of an NO group to prednisolone enhances corticosteroid function following donation of the NO moiety to GR[87]. However, we preliminarily found that peroxynitrite-induced nitration of GR down-regulated GR action (manuscript in preparation). Perhaps different targets of nitration are involved in the difference in GR action.

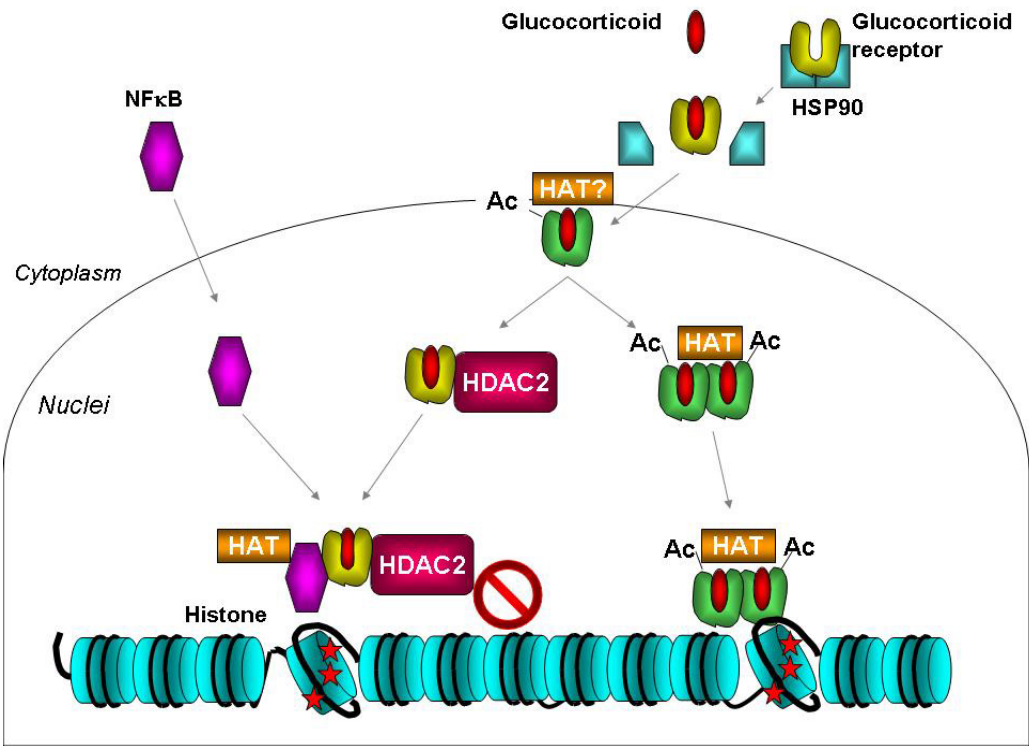

**FIGURE 4.** GR acetylation. After ligand binding, GR is acetylated and dimerized. The dimer binds GRE and induces anti-inflammatory factors where this binding likely causes side effects. On the other hand, HDAC2 is recruited to the acetylated GR and deacetylates it to allow binding to NF-κB, resulting in repression of NF-κB–dependent gene expression.

### Ubiquitinylation/Sumoylation

The ubiquitin-proteasome–dependent protein degradation pathway (UPP) regulates the turnover of many transcription factors including steroid hormone receptors, such as the ER and PR. Glucocorticoid treatment induced a down-regulation of GR[88] and the proteasome inhibitors, MG-132 or a beta-lactone, blocked GR protein down-regulation by the glucocorticoid treatment and also significantly enhanced glucocorticoid-induced transcriptional activation[76,89]. This suggests that the down-regulation of GR is highly controlled by the UPP pathway and GR is a potential substrate for ubiquitinylation. Lys-426 was identified as one target of ubiquitinylation by a mutagenesis study[76] (Fig. 2).

Wallace and Cidlowski indicated that the phosphorylation status of the glucocorticoid receptor plays a prominent role in receptor protein turnover and phosphorylation is a key signal for ubiquitination and proteasomal catabolism of GR[76]. As well as glucocorticoid binding, Kinyamu and Archer showed that the ligand-bound ER regulates GR expression via ubiquitinylation, suggesting that cross-talk between the GR and ER involves multiple signaling pathways, indicative of the mechanistic diversity within steroid receptor-regulated transcription[90]. Thus, the UPP is responsible for the accelerated down-regulation of GR levels in cells subjected to chronic glucocorticoid or estrogen agonist exposure.

Small ubiquitin-related modifier-1 (SUMO-1) is covalently attached to many cellular targets to regulate protein-protein and protein-DNA interactions, as well as localization and stability of the target protein. GR is reported to be post-translationally modified by SUMO-1 (sumoylated) in a ligand-enhanced fashion[91,92] on two sites: on the N-terminal transactivation region (K277, 293) and on the LBD of GR (K703). The two N-terminal sites are the major acceptor sites for SUMO-1 attachment. The SUMO-1-conjugating E2 enzyme Ubc9 is also known to interact with the GR[93]. SUMO-1 modification of GR regulates the stability of the protein and also enhances the ligand-induced transactivation of GR[92]. In contrast, SUMO-1 inhibits the receptor's transactivation potential[91]. Collectively, the role of SUMO-1 on GR is still controversial, but regulates the synergetic control function of GR and serves as a unique signal for activation and destruction.

### MOLECULAR MECHANISMS OF GLUCOCORTICOID RESISTANCE IN AIRWAY DISEASE

As mentioned above, an important feature of severe asthma and COPD is glucocorticoid resistance[1,2]. As well as the defect of controlling lung function by glucocorticoid, and more importantly for understanding the molecular basis of glucocorticoid insensitivity, glucocorticoid-resistant asthma is also associated with impaired *in vitro* and *in vivo* responsiveness of PBMCs to the suppressive effects of corticosteroids on proinflammatory cytokine production[94].

Regarding COPD patients, several large studies suggest that long-term treatment with corticosteroids did not stop the inexorable decline of lung function. This is consistent with the demonstration that inhaled or oral corticosteroids fail to reduce inflammatory cell numbers, cytokines, chemokines, or proteases in induced sputum or bronchial biopsies of patients with COPD[95].

At a molecular level, resistance to the anti-inflammatory effects of glucocorticoids can be induced by several mechanisms. The reduction in corticosteroid responsiveness observed in cells from these subjects has been ascribed to reduced number of GR, altered affinity of the ligand for GR, reduced ability of the GR to bind to DNA, or increased expression of inflammatory transcription factors, such as AP-1, that compete for DNA binding[94].

#### Defects in GR Sequence and Pharmacokinetics

Unlike familial glucocorticoid resistance where there is a mutation in the LBD of GR and a subsequent resetting of the basal cortisol level, corticosteroid-resistant (CR) patients have normal cortisol levels and are not addisonian[96]. Using standard dexamethasone suppression tests, it has been shown that glucocorticoid-resistant severe asthmatics do not have an altered secretory rate of endogenous cortisol or an altered sensitivity of the HPA axis[97]. It is unlikely, therefore, that the defect in CR asthma lies in the structure of the GR.

It has previously been demonstrated, using whole cell binding assays, that no significant changes exist in monocyte and T-cell binding affinity ($K_d$), receptor density, and expression of the GR in patients with glucocorticoid-resistant asthma[98,99] . Furthermore, Sher et al. have described two patterns of ligand binding abnormalities in glucocorticoid-resistant asthmatics termed type 1 and 2[100]. The more common type 1 defect was associated with reduced $K_d$ of GR, normal receptor numbers, and was specific to T cells. The less common type 2 defect was associated with reduced GR receptor density with a normal $K_d$ and was seen in the total mononuclear cell population. These differences were detected only in the nucleus and not the cytoplasm, possibly reflecting an effect of a nuclear protein masking the GR ligand binding site or an altered conformation of the activated GR. The type 1 defect was mimicked by incubation of cells with high concentrations of IL-2 and IL-4 or by IL-13 alone[94,66].

The mechanism of IL-2/IL-4, or IL-13 alone, induced defect of ligand binding characteristics have been explained in two ways. Leung and colleagues have associated these changes with an increased expression of the dominant negative isoform of GR, GRβ[94], although others have been unable to detect enhanced GRβ expression in PBMCs from these glucocorticoid-resistant patients[66,101]. Irusen et al. have recently demonstrated that the effects of IL-2/IL-4 on GR ligand binding and dexamethasone regulation of IL-10 release were blocked by the p38 MAPK inhibitor SB203580[66]. Activation of p38 MAPK by IL-2/IL-4 may result in serine phosphorylation of GR and reduce dexamethasone repression of LPS-stimulated GM-CSF release. These data show that p38 MAPK inhibitors may have potential in reversing glucocorticoid insensitivity and re-establishing the beneficial effects of glucocorticoids in patients with severe asthma. Studies have identified a total of 15 missense, 3 nonsense, 3 frameshift, 1 splice site, and 17 polymorphisms from asthmatic patients, and most of these have been associated with glucocorticoid resistance[63].

Furthermore, Zhou et al. demonstrated at least 7 isoforms of GR by alternative splicing such as GRα, GRβ, GRγ, GR-P, GR-A, and the relative levels of these variants play a role in differential glucocorticoid-induced responsiveness[63], which might be involved in the mechanism of glucocorticoid insensitivity.

### GR Nuclear Translocation and GR/GRE Binding

In one subgroup of severe patients, nuclear localization of GR in response to a high concentration ($10^{-6}$ *M*) of dexamethasone is impaired[50]. The mechanism for this defect is unclear, but may reflect changes in GR phosphorylation[66,102,103].

In a separate subgroup of severe asthma patients, GR nuclear translocation is normal, but dexamethasone cannot correctly stimulate histone H4 lysine (K)5 acetylation[50]. This suggests that glucocorticoids are not able to activate certain genes that are critical to the anti-inflammatory action of high doses of corticosteroids. The mechanism for this effect is unknown, but may be caused by the defect of K5 specific HAT or induction of K5 specific HDAC.

### Cross-Talk with Other Transcription Factors

Transcription factors, especially AP-1, were reported to be excessively activated in glucocorticoid-resistant asthma, in addition to a reduced ability of GR to interact and repress AP-1 activity[104]. Overexpression of c-Fos induced by stimulation of PBMCs derived from mild asthma subject to phorbol myristate acetate (PMA) for 6 h, attenuated the ability of these cells to induce GR-GRE binding after 1-h dexamethasone treatment. These results suggested that excessive AP-1 (c-Fos) under basal conditions is associated with GR to prevent GR function.

### Neutrophilic Inflammation

Neutrophils have been implicated in the pathogenesis of many diseases including COPD, severe asthma, psoriasis, and a variety of collagen-vascular diseases[105,106]. These cells are less sensitive to glucocorticoids than other white blood cell types. The glucocorticoids beclomethasone, budesonide, dexamethasone, fluticasone propionate, hydrocortisone, and prednisolone inhibit apoptosis in a concentration-dependent manner as assessed by flow cytometric analysis, annexin-V binding, and morphological analysis[107]; the maximal inhibition of apoptosis is 50–60%. This is in contrast to T cells and eosinophils, where glucocorticoids induce apoptosis[108,109]. Moreover, glucocorticoids slightly enhance the inhibitory effect of GM-CSF on neutrophil apoptosis. This indicates that glucocorticoids prolong human neutrophil survival by inhibiting apoptosis at clinically relevant drug concentrations via an effect on GR. The mechanisms by which human T cell, eosinophil, and neutrophil apoptotic responses to glucocorticoids differ are unknown. The ratio of GRβ to GRα has been reported to be 73-fold greater in neutrophils than in PBMCs[110]. This reflects differences observed in relative abundance of the mRNAs. GRα/GRβ heterodimers have only 15–20% of the transactivating activity of GRα homodimers. In these studies, the prosurvival activity of dexamethasone was associated with elevated GRβ in freshly isolated neutrophils, perhaps through formation of GRα/GRβ heterodimers.

Formation of 52- and 30-kD GR fragments due to proteolysis by neutrophil elastase (a 28-kD serine protease) is found in cytosol of leukemia cells[111]. Receptor fragmentation in the cytosol is inhibited by methoxysuccinyl-alanyl-alanyl-prolyl-valyl-chloromethylketone, a highly specific inhibitor of neutrophil elastase. The addition of as few as 5% neutrophils to a lymphoid cell suspension provides sufficient elastase to produce receptor fragmentation.

### Latent Viral Infection

Epidemiologic studies have implicated childhood respiratory infection as an independent risk factor for the subsequent development of persistent asthma and COPD[112]. Excess amounts of adenoviral E1A DNA have been described in patients with COPD[113] with the E1A protein expressed in the epithelial cells lining the bronchi, the bronchial glands, the gland ducts, the cells lining the bronchioles, and the type 2 cells lining the alveolar surface[114]. E1A-transfected airway epithelial cells after LPS exposure produce an excess amount of IL-8 and intercellular adhesion molecule (ICAM)-1. Latent adenovirus infection reduces the inhibitory effects of glucocorticoids on airway inflammation in ovalbumin-sensitized guinea pigs[115]. The mechanism is not yet fully understood, though excessive AP-1 induced by adenovirus infection may be involved in steroid resistance[116]. Thus, adenoviruses appear to persist as latent infection in the airways of patients with COPD and adenoviral E1A proteins capable of activating host transcription factors and amplifying host gene expression, including those involved in cigarette smoke–induced lung inflammation and COPD, may contribute to steroid-insensitivity in COPD. More recently, it has been proposed that rhinoviral infection can prevent GR nuclear translocation via an NF-κB-mediated process[1].

### Reduced HDAC Activity in COPD and Severe Asthma

We have demonstrated that in peripheral lung tissue and alveolar macrophages, there is a reduction in HDAC activity and HDAC2 expression in normal smokers, and a striking reduction in patients with COPD[82]. We also demonstrated that HDAC activity is correlated with increased release of the inflammatory proteins TNF-α and IL-8, and a reduction in the inhibitory effect of the glucocorticoid dexamethasone on the expression of these cytokines[81] fig. 5. In fact, HDAC2 knock down in culture cells and sputum macrophages from healthy volunteers showed glucocorticoid insensitivity on LPS-induced GM-CSF release[61]. Furthermore, HDAC2 overexpression in alveolar macrophages from patients with COPD restored glucocorticoid sensitivity, suggesting, HDAC2 is the prerequisite molecule for GR action.

### Oxidative Stress in COPD

Exhaled markers of oxidative stress, such as 8-isoprostane and ethane, are increased in normal smokers with a much greater increase in patients with COPD, even when they have stopped smoking[85]. Oxidative stress and cigarette smoking is reported to activate transcription factors, increase histone acetylation, and switch on inflammatory gene transcription[117,118]. Even at lower dose, we also demonstrated that oxidative stress impairs the function of HDAC2, resulting in resistance to glucocorticoids. Specifically, we showed that tyrosine nitration of HDAC2 impaired its activity[119]. In addition, phosphorylation of HDAC2 via phosphoinositide 3 kinase (PI3K) activation is also a target for reduction of enzyme activity.

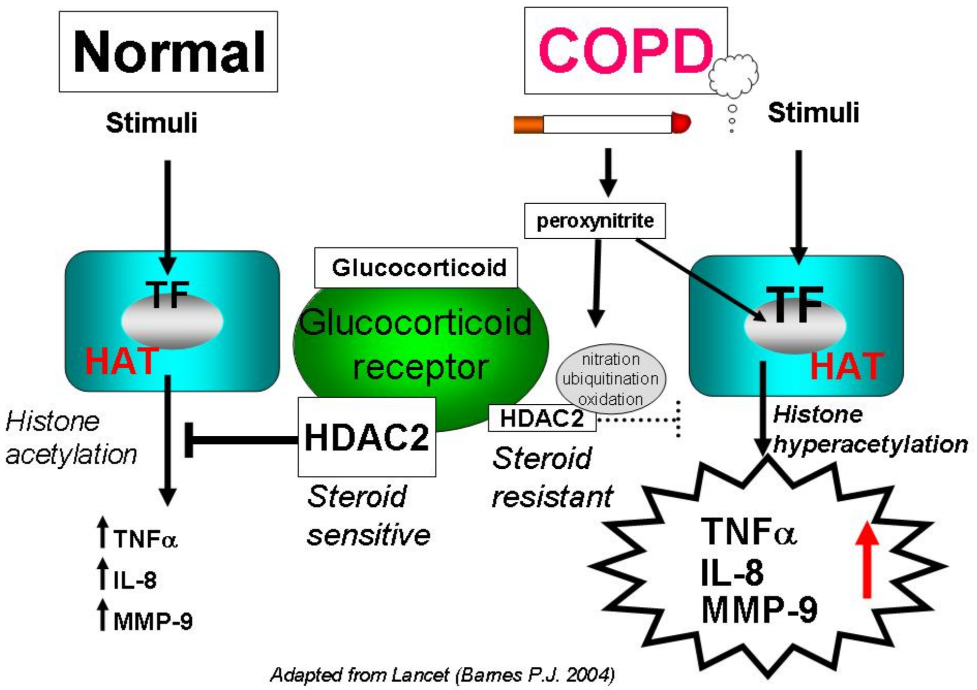

**FIGURE 5.** Proposed mechanism of glucocorticoid insensitivity in COPD patients. In normal alveolar macrophages, NF-κB and other transcription factors are activated following stimulation, and switch on HAT, leading to histone acetylation and, subsequently, to the transcription of genes encoding inflammatory proteins, such as TNF-α, IL-8, and MMP-9. Glucocorticoids reverse this by binding to GR and recruiting HDAC2. This reverses the histone acetylation induced by NF-κB and switches off the activated inflammatory genes. However, in COPD patients, cigarette smoke activates macrophages, but oxidative stress (acting through the formation of peroxynitrite) impairs the activity of HDAC2. This amplifies the inflammatory response to NF-κB activation, but also reduces the anti-inflammatory effect of glucocorticoids, as HDAC2 is now unable to reverse histone acetylation. This figure is adapted from Barnes et al.[29].

### THERAPEUTIC IMPLICATION

Inhaled glucocorticoids are now used as first-line therapy for the treatment of persistent asthma in adults and children in many countries, as they are the most effective treatments for asthma currently available[120]. However, at high doses, systemic absorption of inhaled glucocorticoids may have deleterious effects, even when patients with COPD and severe asthma did not respond to treatment. So there has been a search for safer glucocorticoids or compounds to restore glucocorticoid sensitivity.

#### Dissociated Corticosteroids

There is an ongoing search for novel glucocorticoids that selectively *trans*repress without significant *trans*activation. The major task in developing these drugs is to dissociate the anti-inflammatory effects from the endocrine actions that are associated with side effects.

Recently, a novel class of glucocorticoids has been described in which there is potent *trans*repression with relatively little *trans*activation. These “dissociated” glucocorticoids, including RU24858, RU40066, and ZK216348, have anti-inflammatory effects *in vitro*, although there is little or some separation of anti-inflammatory effects and systemic side effects *in vivo*[9,121,122,123]. Several nonsteroidal selective glucocorticoid receptor agonists (SEGRA) have recently been reported that show dissociated properties in human cells[124,125]. Several of these dissociated glucocorticoids and SEGRA are now in clinical development and show good separation between *trans*repression and *trans*activation actions. This suggests that the development of dissociated glucocorticoids and SEGRA is possible with a greater margin of safety and may even lead to the development of oral compounds that do not have significant adverse effects.

### Restoration of Glucocorticoid Action

As discussed above, oxidative stress is markedly increased in severe asthma and in patients with COPD, and can attenuate GR function via reduction of HDAC2 or excessive activation of transcriptional factors. Since oxidative and nitrative stress may drive the glucocorticoid insensitivity, antioxidants such as N-acetyl cysteine (NAC), fudostein, or inhibitors of inducible NO synthase (NOS2) should reverse glucocorticoid resistance. A couple of clinical studies with NAC in patients with COPD have produced variable results[126,127,128]. These studies did not formally examine glucocorticoid responsiveness in these patients although antioxidants restore glucocorticoid sensitivity via restoration of HDAC activity *in vitro*. Activation of HDACs is a more direct approach and may have therapeutic potential. Theophylline has been shown to have this property, resulting in marked potentiation of the anti-inflammatory effects of glucocorticoids[129]. *In vitro* theophylline at therapeutic concentrations is able to restore the HDAC activity reduced by oxidative stress and restore glucocorticoid responsiveness[130]. This action of theophylline is not mediated via phosphodiesterase inhibition or adenosine receptor antagonism and, therefore, appears to be a novel action of theophylline[129] or its PI3Kδ inhibition[131]. It may be possible to discover similar drugs that could form the basis of a new class of anti-inflammatory drugs without the side effects that limit the use of theophylline.

As well as HDAC reduction, defect of GR nuclear translocation is another important impairment of GR action. We recently showed that long-acting $\beta_2$-agonist (LABA) is more effective in these patients. This combination therapy of LABA and glucocorticoid is established as a more effective therapy in asthma and also in COPD[132]. Preliminary *in vitro* and *in vivo* data suggest that the combination of long-acting inhaled $\beta_2$-agonists and inhaled glucocorticoids may have a synergistic effect[133]. In the *in vitro* studies, LABAs have been shown to induce GR nuclear translocation and enhance GR DNA binding in the absence of ligand[134]. More recently, Usmani et al. demonstrated synergic activation of GR by LABA[135]. Other compounds, such as phosphodiesterase (PDE) inhibitors and leukotriene antagonists, were also reported to enhance glucocorticoid action and reduce the use of glucocorticoid, but the molecular mechanism is not fully evaluated[136,137,138].

### Nonsteroidal Anti-Inflammatory Treatments: Glucocorticoid-Sparing Therapy

A variety of anti-inflammatory compounds are now being developed. Many of the anti-inflammatory effects of glucocorticoids appear to be mediated via inhibition of the transcriptional effects of NF-κB, and small-molecule inhibitors of IKK (inhibitor of I-κB kinase-2) that activate NF-κB are in development[139]. p38 MAPK inhibitors and PI3K inhibitors also have therapeutic potential as glucocorticoid-sparing agents[16,140] because they are expected to affect neutrophils, which are insensitive to glucocorticoids, as well as the normal profile of anti-inflammation. Macrolides are recognized as anti-inflammatory compounds[141,142] and macrolide-steroid conjugates are now under development (previous PLIVA Research Institute).

## CONCLUSION

Current systemic and inhaled pharmacological treatment of severe asthma and COPD is unsatisfactory as it does not significantly influence the severity of the disease or its natural course apart from the treatment of exacerbations.

The identification of an active resistance mechanism suggests that glucocorticoid resistance/insensitivity is potentially reversible, which would have enormous implications for the future therapy of chronic inflammatory diseases. Since oxidative and nitrative stress may inactivate HDAC2, interfere with the action of other HDACs, alter cofactor recruitment, or induce post-translational modification of GR, antioxidants, HDAC activators, and kinase inhibitors are likely to prove effective in restoring corticosteroid sensitivity in these diseases.

### ACKNOWLEDGMENTS

We regret that, owing to space constraints, we were not able to cite all the important original work in this field, and apologize to those authors whose work we have not cited. The authors would like to acknowledge funding from The Wellcome Trust, The Medical Research Council, AstraZeneca, GlaxoSmithKline, and Mitsubishi.
